# Eosinophil Cationic Protein (ECP), a predictive marker of bullous pemphigoid severity and outcome

**DOI:** 10.1038/s41598-017-04687-5

**Published:** 2017-07-06

**Authors:** Delphine Giusti, Gregory Gatouillat, Sébastien Le Jan, Julie Plée, Philippe Bernard, Frank Antonicelli, Bach Nga Pham

**Affiliations:** 1 Laboratory of Dermatology, Faculty of Medicine of Reims, EA 7319, IFR 53, University of Champagne-Ardenne, Reims, France; 2 Laboratory of Immunology, Reims University Hospital, University of Champagne-Ardenne, Reims, France; 3 Department of Dermatology, Reims University Hospital, University of Champagne-Ardenne, Reims, France

## Abstract

Bullous Pemphigoid (BP) is an inflammatory rare autoimmune bullous dermatosis, which outcome cannot be predicted through clinical investigations. Eosinophils are the main immune infiltrated cells in BP. However, the release of Major Basic Protein (MBP), Eosinophil Derived Neurotoxin (EDN), and Eosinophil Cationic Protein (ECP) upon eosinophil activation has still not been evaluated with respect to BP development. MBP, EDN and ECP were measured by ELISA in serum (n = 61) and blister fluid (n = 20) of patients with BP at baseline, and in serum after 2 months of treatment (n = 41). Eosinophil activation in BP patients was illustrated at baseline by significantly higher MBP, EDN and ECP serum concentrations as compared with control subjects (n = 20), but without distinction according to disease severity or outcome. EDN and ECP values were even higher in the blister fluids (*P* < 0.01 and *P* < 0.05, respectively), whereas MBP values were lower (*P* < 0.001). ECP serum concentration decreased after 60 days of treatment in BP patients with ongoing remission but not in patients who later relapsed (*P* < 0.05). A reduction of at least 12.8 ng/mL in ECP concentrations provided a positive predictive value for remission of 81%, showing that ECP serum variation could be a useful biomarker stratifying BP patients at risk of relapse.

## Introduction

Bullous pemphigoid (BP) is a rare autoimmune blistering skin disease, with autoantibodies targeting the hemidesmosome proteins BP180 and BP230^1–3^. The BP disease preferentially affects the elderly^4, 5^. In this autoimmune and inflammatory disease, superpotent topical corticosteroids have proven their efficacy and therefore are currently used as the first line of treatment in France^6, 7^. Nevertheless, clinical relapse despite therapy is observed within the first year of treatment in approximately 30% of patients with BP^8^, and so far, no single biomarker of disease outcome in BP warrants wide acceptance emphasizing the need for future investigation of biomarkers in large-scale longitudinal studies.

In patients with BP, variations in inflammatory cytokine expression has been shown to play a critical role in the pathological processes related to the outcome of the disease^9, 10^. In this line, BP is clinically characterized by the presence of tense blisters along with intense pruritus, occurring preferentially on inflammatory, erythematous plaques. The use of mouse models allowed the demonstration that binding of autoantibodies onto their antigenic proteins is associated with an inflammatory cascade, including mast cell degranulation, and the recruitment and activation of neutrophils leading to dermal-epidermal separation^1, 11–13^. However, the role of eosinophils, which are the main tissue-infiltrated inflammatory cell type in the lesional skin of BP patient^14^, and especially how their granule proteins could impact BP disease severity and outcome still remains unclear in human.

Indeed, eosinophil implication in BP pathophysiological process is still not well defined. Eosinophils are endowed with a potent army of proinflammatory mediators including basic proteins stored in eosinophil granules, as well as cytokines, chemokines, lipid mediators, various proteases and components of the oxygen burst^15, 16^. Although this cytotoxic capacity is considered important in the immune response to infection with bacteria, parasites, viruses and tumor cells, their inappropriate accumulation can cause severe tissue damage in a wide variety of diseases affecting the skin but also the lungs, the heart, or the gastrointestinal tract^16^. Specific granules of human eosinophils contain robust store of preformed basic proteins. The crystalloid core of the secondary granules is constituted by the highly cationic eosinophil Major Basic Protein (MBP) and is covered by a matrix composed, amongst others, by the 2 ribonucleases, Eosinophil Cationic Protein (ECP) and Eosinophil Derived Neurotoxin (EDN)^17, 18^. As a result of eosinophil activation, MBP, ECP and EDN are released in the extracellular compartment.

The aim of this work was to study eosinophil activation in BP by measuring MBP, EDN and ECP concentrations according to the disease extent and disease outcome. At the time of diagnosis, the concentrations of these 3 granule proteins were evaluated both in the serum and the blister fluid of patients with BP with respect to those determined in the serum of healthy subjects. Variations of MBP, EDN and ECP serum concentrations between baseline and after 2 months of treatment were analyzed according to both disease extent at the time of diagnosis and disease outcome during the first year of follow-up.

## Results

### Characteristics of patients

The mean age of the 61 included BP patients at diagnosis was 80.6 years and the sex ratio F/M was 1.9. At baseline, 29 (47.5%) patients had an extensive disease and 32 (52.5%) had a moderate disease. During the 1-year follow-up, 10 (16.4%) patients died and 3 (4.9%) were lost to follow-up. All the living patients achieved disease control after 2 months under treatment, while 16 (33%) experienced at least one relapse after this initial disease control within the 1-year follow-up. Relapse occurred preferentially in patients with extensive disease at the time of diagnosis (57% in extensive disease *vs* 29% in the moderate disease group) (*P* = 0.03 Pearson’s Chi-squared test). The characteristics of the 41 patients, for who variations in serum concentrations of the 3 granule proteins MBP, ECP, EDN between days 0 and 60 could be analyzed, were similar to the whole studied population defined above.

### Serum analysis of MBP, EDN, ECP

**Figure 1:**
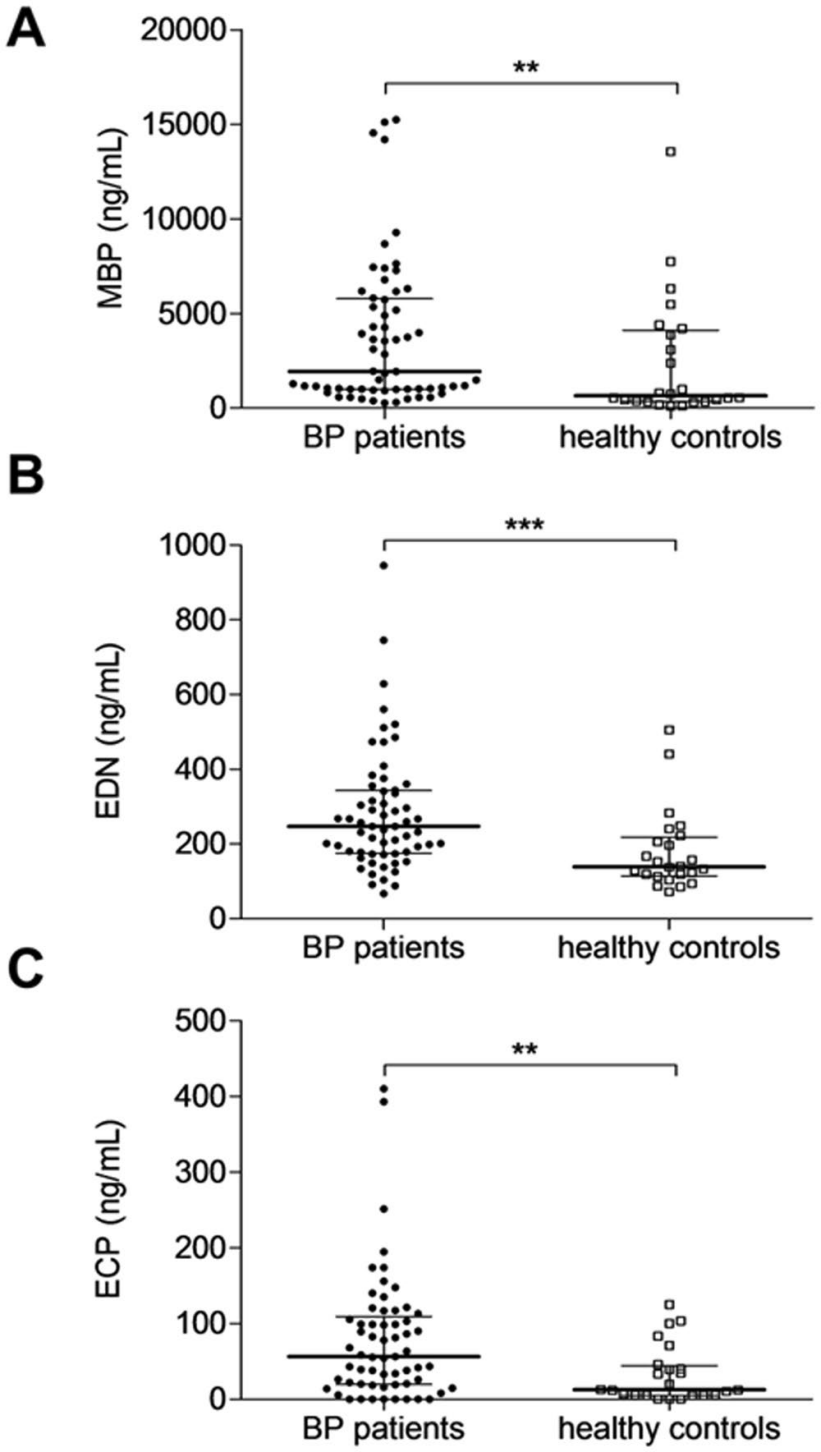
Serum concentrations of Eosinophil granule proteins at baseline. Major Basic Protein (MBP) (**A**), Eosinophil Derived Neurotoxin (EDN) (**B**) and Eosinophil Cationic Protein (ECP) (**C**) serum levels from 61 patients with active bullous pemphigoid and 24 healthy control subjects were measured by specific ELISA at the time of diagnosis. Results are expressed as ng/mL. (**P < 0.01; ***P < *0.001*). Lines correspond to median and interquartile ranges.

Serum concentrations of eosinophil granule proteins were measured at the time of diagnosis in 61 BP patients and 24 age- and sex-matched control subjects. At baseline, MBP serum concentration was significantly higher in BP patients than in control subjects (mean ± standard deviation 3725 ± 3854 *vs* 2399 ± 3271 ng/mL, *P* = 0.0066 Mann Whitney test). EDN serum concentration also was significantly higher in BP patients than in control subjects (282 ± 162 ng/mL *vs* 178 ± 107 ng/mL, *P* = 0.0004 Mann Whitney test). The same type of results was observed when measuring ECP (78 ± 82 in patients with BP *vs* 33 ± 37 ng/mL in control subjects, *P* = 0.0057 Mann Whitney test) (Fig. 1).

At the time of diagnosis, we next evaluated whether the concentrations of eosinophil granule proteins were linked either to disease extent or to disease outcome. With respect to disease extent, no significant differences were noticed when concentrations of MBP, EDN, ECP measured in the serum of patients with moderate BP disease were compared with those measured in the serum of patients with extensive BP disease. Also, no significant differences were noticed with respect to disease outcome, when the serum concentrations of MBP, EDN, and ECP were compared between patients with BP with ongoing remission and those who experienced a relapse during the first year of follow up (Table 1).

**Table 1:** MBP, EDN and ECP serum concentrations in patients with BP at the time of diagnosis according to either the extent (A) or the outcome of the disease (B).

A			
Disease extent	Moderate (n = 24)	Extensive (n = 19)	*P* value
**Protein concentration (ng/mL)**			
MBP	2722 (±3362)	4573 (±5142)	0.44
EDN	327 (±181)	332 (±159)	0.62
ECP	90 (±117)	52 (±45)	0.82
**B**			
**Disease outcome**	**Remission (n = 26)**	**Relapse (n = 17)**	***P*** **value**
**Protein concentration (ng/mL)**			
MBP	3250 (±4092)	3983(±4664)	0.51
EDN	336 (±193)	319 (±130)	0.86
ECP	85 (±114)	56 (±49)	0.91

Extensive disease was defined as more than 10 new blisters daily. Relapse was defined as the reappearance of at least 3 new daily blisters in between one year follow-up. No statistically significant difference was observed using Mann Whitney test.

**Figure 2:**
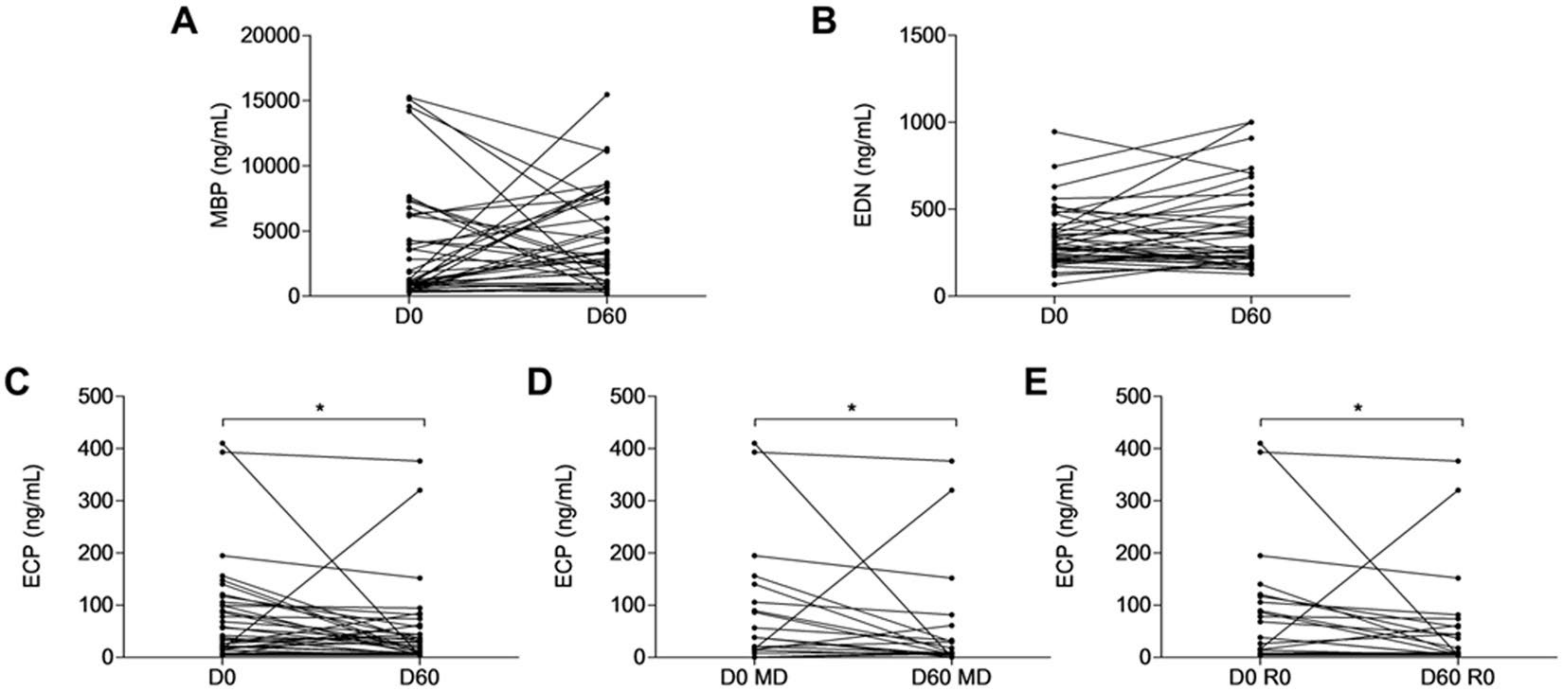
MBP, EDN and ECP serum variations between baseline and day 60. Variations in the serum concentrations of MBP (**A**), EDN (**B**) and ECP (**C**) were measured by mean of specific ELISAs between days 0 and 60. (MD: Mild disease; R0: Remission). (*P < 0.05).

**Figure 3:**
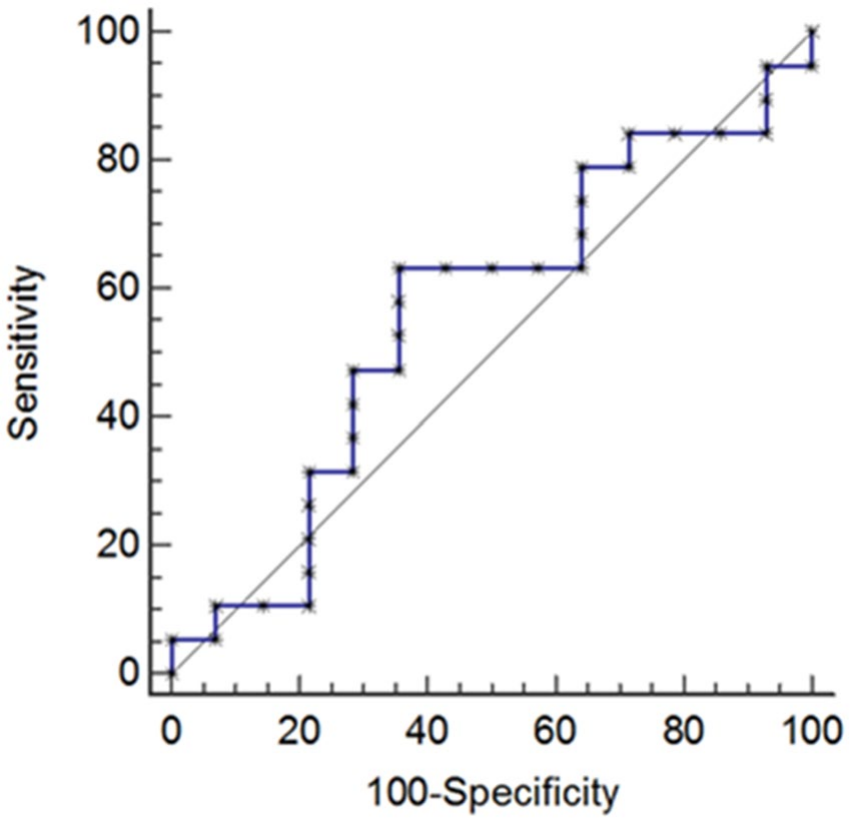
Prognostic value of the decrease in ECP serum concentrations between days 0 and 60. The prognostic properties of the decrease of ECP serum concentrations measured by means of ELISA between baseline and day 60 with respect of the occurrence of a clinical remission in patients with BP are depicted as a receiver operating characteristic (ROC) curve.

A complete clinical and biological follow-up was achieved in 41 BP patients, at least 60 days after the initiation of treatment. Therefore, we evaluated the variation of the 3 eosinophil granule proteins serum concentrations between the time of diagnosis and after 60 days of therapy. Statistical analysis using the Wilcoxon matched pairs test showed that the concentration of MBP remained elevated in the serum of patients with BP after 60 day treatment (3664 ± 4355 *vs* 4236 ± 3607 ng/mL, *P* = 0.30) (Fig. 2A). Subgroup analyses also showed no significant variation of MBP serum concentrations between days 0 and 60 according to both disease extent or disease outcome (Table 2). Variations in EDN serum concentrations did not demonstrate significant differences after treatment either (Fig. 2B). In contrast, upon treatment, ECP serum concentrations significantly decreased after 60 days of therapy (69 ± 91 *vs* 45 ± 76 ng/mL, *P* = 0.011 Wilcoxon matched pairs test) in the whole population of patients with BP (Fig. 2C). Subgroup analysis revealed that the observed ECP serum concentration variations within this period of time were related to disease extent. Actually, ECP concentrations decrease between days 0 and 60 was observed in the serum of patients with moderate BP disease (*P* = 0.040, Wilcoxon matched pairs test) (Fig. 2D), but not in the serum of patients with extensive BP disease. Variation in ECP serum concentrations was also related to BP disease outcome, as the decrease in ECP concentrations between days 0 and 60 was significant in patients with BP who achieved remission (*P* = 0.0503, Wilcoxon matched pairs test) (Fig. 2E), but not in patients with BP who relapsed during the first year of treatment. A ROC curve was calculated to determine a cutoff value of ECP serum concentration decrease for the prediction of clinical remission. The cutoff value of 12.8 ng/mL corresponding to a decrease of ECP concentration between baseline and day 60 provided 63.2% sensitivity, 64.3% specificity, 81.1% positive predictive value, and 41.8% negative predictive value for the occurrence of clinical remission of BP (Fig. 3).

**Table 2:** Variations of MBP, ECP and EDN serum concentrations in patients with BP according to their clinical outcome.

	Disease outcome	Day 0	Day 60	*P* value
MBP (ng/mL)	Remission (n = 26)	3341 ± 4150	3848 ± 3142	0.415
	Relapse (n = 17)	4169 ± 4751	4844 ± 4272	0.587
EDN (ng/mL)	Remission (n = 26)	338 ± 197	395 ± 262	0.162
	Relapse (n = 17)	320 ± 135	336 ± 193	0.897
ECP (ng/mL)	Remission (n = 26)	80 ± 110	54 ± 95	**0.050**
	Relapse (n = 17)	56 ± 49	31 ± 27	0.132

ECP serum concentrations were significantly decreasing between baseline and day 60 in patients with BP who achieved clinical remission.

### Blister fluid analysis of MBP, EDN and ECP

**Figure 4:**
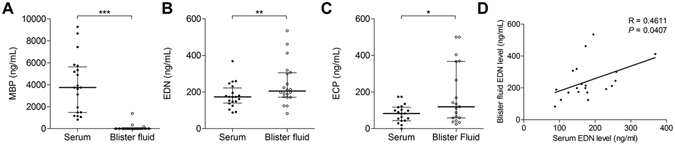
Comparison of serum and blister fluid concentrations for MBP, ECP and EDN in 20 patients with active bullous pemphigoid. The serum blister fluids concentrations of MPB (**A**), EDN (**B**) and ECP (**C**) were measured by means of specific ELISAs. Variations between serum and blister fluids were analyzed by means of Wilcoxon test for MBP and EDN and paired t test for ECP. Correlation between EDN concentrations in the serum and in the blister fluids was performed by means of Spearman’s correlation coefficient analysis. Statistical significance was as follow: (*P < 0.05, **P < 0.01; ***P < *0.001*). Lines define median and interquartile ranges.

The concentrations of MBP, EDN and ECP in the blister fluid harvested from 20 BP patients at the time of diagnosis were compared with those in the respective serum samples (Fig. 4). ECP and EDN blister fluid concentrations were significantly higher than their respective serum concentrations (*P* = 0.011 and *P* = 0.0095, respectively, Wilcoxon matched pairs tests), whereas MBP concentration was undetectable within most of the blister fluids tested (80%) (Fig. 4A–C).

We then studied the relation between the granule protein production in the skin and the relative serum levels. No correlation could be drawn between the ECP concentrations within the blister fluid and in the serum (R = 0.34, *P* = 0.15) (data not shown). In contrast, we found that the EDN concentrations measured in the blister fluids were correlated to those determined in the respective serum samples (R = 0.46, *P* = 0.04 Spearman Correlation) (Fig. 4D).

Hence, the 3 eosinophil granule proteins displayed 3 different profile patterns: i.e. MBP demonstrated undetectable blister fluid concentrations despite high serum concentrations; EDN demonstrated high blister fluid concentrations in correlation with high serum concentrations; and ECP demonstrated high blister fluid concentrations but without correlation with the respective serum concentrations.

## Discussion

Highlighting the importance of eosinophils activation in the pathogenesis of BP, we showed in this study that each of the 3 granule proteins MBP, EDN and ECP displayed specific profile pattern according to either body distribution, to disease extent at the time of diagnosis and to disease outcome, therefore suggesting that these proteins should endow specific roles during BP disease development.

Eosinophil activation was illustrated by elevated concentrations of MBP, EDN and ECP in the serum of patients with BP at baseline compared with the level observed in age-matched control subjects, supporting a previous study conducted on a smaller population of patients with BP^19^. In this line, it has been shown that the level of the eosinophil activator IL-5 was increased in the serum of patients with BP as compared with healthy subjects^20, 21^. Activated CD4^+^ T cells, monocytes, mast cells and eosinophils themselves express IL-5^22^. Interestingly, it has been shown that autoreactive CD4^+^ T cell clones responding to BP180 autoantigen secrete IL-5^23^, potentially creating an amplification loop as this cytokine induces stimulated B cells to differentiate into plasmocytes^22^. However, if such an activation process may occur in patients with BP, the serum expressions of these 3 eosinophil granule proteins at baseline were not related to either disease extent or disease outcome. Then, further investigations are required to better understand the implication of these proteins in the pathophysiological process associated to BP.

At the time of diagnosis, the concentrations of the 3 eosinophil granule proteins were all elevated in the serum of BP patients, but showed different profile patterns in their respective blister fluid. As the eosinophils are known to be recruited from the circulation into inflammatory foci^24, 25^, the best reflect of their activation should be found locally in BP lesions. Indeed, the concentration of ECP and EDN were higher in the blister fluid than in the serum. However, MBP was undetectable by ELISA in the blister fluids. Immunofluorescence performed on paraffin embedded sections of lesional skin of BP patients reinforced our result by showing that ECP and EDN were much more expressed than MBP by eosinophils at the site of skin lesion (data not shown). Furthermore, only EDN concentrations in the blister fluid and in the serum were correlated, suggesting that the concentrations of this protein were somehow connected. Thus, the three eosinophil granule proteins displayed three different profile patterns, suggesting that subtle regulatory mechanisms induced by the local environment may control the production of each of these proteins. The differences observed between MBP, EDN, and ECP expression in the serum and in the involved skin may be explained by the mechanisms of eosinophil granule release upon stimulation. The activation of the eosinophils causing the release of these inflammatory mediators can occur after various stimuli: either following direct interaction with integrin expressed by resident cells or ligation of eosinophil Fcγ receptor or even following eosinophils priming by eotaxin, IL-5 or platelet activating factor. According to the type of stimulus, selective release of eosinophil granule protein may be induced^26^. The blister skin environment seems to be favorable in patients with BP to selective ECP and EDN release by activated eosinophils. Therefore it is of importance to deeper defining the activation network to better understand the pathophysiological mechanisms involved in eosinophils activation in BP disease.

Elevated concentrations of EDN and ECP in the blister fluid support the hypothesis that these two basic ribonucleases may contribute to tissue damage and blister formation. Indeed, EDN and ECP can disrupt skin integrity and cause inflammation because of their ribonuclease activity and of their cationic charge, as it was evidenced in animal model^27^. Actually, intradermal injection of EDN and ECP to guinea pig skin led to ulcerated or crusted lesions with marked cellular infiltration that persisted over a period of 6 weeks^27^, suggesting that these proteins actively participate to skin lesions in BP. Conversely to EDN and ECP, we did not detect MBP in the blister fluids of patients with BP, and therefore did not confirm a previous observation performed on a small series of skin biopsy specimens^19^. With respect to BP disease, and to the large amount of proteases within the blister fluids, we cannot exclude that the differences observed in MBP concentration may be related to different half-life or sensibility to proteolysis in the serum and in the blister fluid, although further investigations will be required to prove such a hypothesis. Also, physical-chemical properties (MW, pHi) could also interfere in the expression and localisation of these eosinophil markers., The discrepancies between our results and those of Borrego and colleagues^19^ could be related to sampling analysis. Indeed, intradermal injection of MBP into guinea pig skin did not induce skin ulcerated lesions like ECP end EDN, but erythema and induration^27^, therefore suggesting that MBP could interfere in the early prebullous phase in the pathogenic process of BP. Furthermore, in line with the lack of MBP detection in the blister fluids, a negative correlation between the serum concentration of MBP at baseline and disease severity (BPDAI) (R = −0.57, P = 0.008 Spearman correlation) was observed, which is in setting with a prebullous production of this protein. Such a sequential release of MBP versus EDN and ECP is in setting with specific eosinophil activation networks for each of these 3 granule proteins. For instances, it was shown that MBP is generally released after IgE complex challenging^28^. However, it must be noted that although MBP could interfere in the early erythematous phase of BP, the concentration of this protein did not decrease over time upon treatment, and this although the disease was controlled in all patients with BP. Thus, further studies are required to determine why MBP would be involved in the early erythematous phase but not later, and why the concentration of this protein is not controlled under corticosteroid treatment.

The longitudinal study revealed that only evaluation of ECP serum concentrations demonstrated a clinical relevance. Indeed, ECP concentrations decreased under treatment in the serum of patients with BP with ongoing remission but not in those of patients with BP who experienced a relapse during the first year of follow-up. In this line, we also found that ECP serum concentrations decreased in patients with moderate BP disease, but not in those of patients with severe disease, therefore completing a previous study demonstrating that the elevated serum concentrations of ECP decreased upon immunosuppressive treatment in patients with BP^29^. Such a specific profile with respect to disease outcome was not observed either with MBP or EDN, for which serum concentrations remained elevated in all patients with BP. Thus, the variation in ECP concentration according to disease severity and especially to disease outcome is in line with our previous publications, showing (i) that decrease in anti-BP180 serum concentrations were useful to predict BP outcome^8^, and (ii) a sustained or even increased concentrations of IL-17 and IL-23 in those patients who experienced a relapse under treatment^10^. Although both decrease of ECP concentration and that of anti-BP180 antibody concentration were more pronounced in the serum of patients with BP with ongoing remission, no correlation was observed between these 2 molecules (data not shown). In addition, sustained concentrations of ECP, IL-17, IL-23 and CXCL10 in the serum of patients with BP who later relapse showed that patients with BP at risk of relapse have an implicit pathologic inflammatory background which persisted under therapy. Like for anti-BP180 autoantibodies and IL-23 concentrations^9, 10, 30^, we evidenced that a decrease in ECP serum concentration of 12.8 ng/mL or more, between days 0 and 60, was associated with a positive predictive value (>80%) for BP clinical remission. Noteworthy, the specificity of 64% defined from the ROC curve is relatively low certainly due to inter-individual variability. Consequently, the cut off value may be difficult to use in routine practice. Nevertheless with the different ECP serum profiles displayed by BP patients with and without relapse, these results demonstrate that serum ECP variations are either involved in the BP physio-pathological mechanisms or representing biological marker of BP outcome.. Therefore, quantification in the concentration of diverse molecules further supports the idea that the constitution of an inflammascore (IL-17, IL-23 and ECP) could be useful in predicting the likelihood of relapse in patients with BP.

In conclusion, we demonstrated that eosinophils are activated in skin lesions, with high concentrations of ECP and EDN evidenced in the blister fluid of BP patients. The specific kinetics of the 3 eosinophils proteins also strengthened the evidence for a critical pathogenic role of eosinophils in BP disease process although further studies are still required to fully understand the role of eosinophil activation associated with the different phases of blister formation.

## Material and Methods

### Study Patients and Design

This prospective, multicenter, observational study was conducted in 8 French dermatology departments among which 3 belong to the French Referral Center for Autoimmune Bullous Diseases. Sixty-one patients with BP were included between November 2009 and November 2013.

Consecutive patients with newly diagnosed BP were included based on the presence of at least 3 out of 4 well established clinical criteria by Vaillant *et al*. in combination with positive direct immunofluorescence findings (linear deposition of IgG and/or C3 along the basement membrane zone)^31^. Two groups were distinguished at the time of inclusion based on the daily blister count: a group of patients with moderate BP disease (less than 10 new blisters per day) and a group of patients with extensive BP disease (more than 10 new blisters per day). All of these patients with BP were followed during one year for clinical outcome assessment. Relapse was defined as the reappearance of at least 3 new daily blisters along with pruritus and/or erythematous, eczematous or urticarial plaques. Superpotent topical corticosteroids (clobetasol) were the first line treatment as it is currently recommended in France^6, 32^. Among the 61 patients included, 41 were enrolled in the dermatology department of Reims University Hospital, for who serum samples were also collected after 2 months treatment.

### Ethics statement

The study was approved by the Ethic Committee of the University Hospital of Reims (institutional review board; 14.04.2009), and all of the subjects gave their informed and written consent before participating in the study in accordance with the Helsinki Declaration. The methods were carried out in accordance with the relevant guidelines and regulations.

### Eosinophil granule proteins analysis in sera and blister fluids

Sera were collected at baseline and day 60 after patient care. Blister fluids were collected from 20 patients with BP at baseline in one center (University Hospital of Reims) without anticoagulant. The samples were aliquoted and stored at −80 °C. MBP, EDN and ECP concentrations were measured by means of commercially available enzyme linked immunoassays (Cloud-Clone Corporation, Houston, Texas), in the serum and in the blister fluids in the same experiment, with respect of the instructions provided by the manufacturer. First dilution factor was 1:50 for MBP and EDN and 1:100 for ECP. Further dilutions were performed for samples over-range of calibration (1:100 and 1:200 for MBP and EDN, 1:200-1:400 for ECP). Serum levels of patients with active BP were compared with the concentration in the serum of 24 control subjects obtained from age- and sex- matched patients without inflammatory or auto-immune disease hospitalized at the University Hospital of Reims.

### Statistical analysis

For statistical analysis, values distribution was assessed using D’Agostino and Pearson omnibus normality test. Mann Whitney test was used for comparison of MBP, ECP, EDN concentrations in the serum of patients with BP and in the serum of control subjects at baseline. The Wilcoxon and paired t-test were used for comparison analysis of both serum proteins levels between baseline and day 60, and between serum and blister fluid proteins levels. Correlations among ECP and EDN blister fluid levels were performed by Spearman’s correlation coefficient. *P* ≤ 0.05 was considered as statistically significant.
